# Phenotypic Variation Characterization and Multiple-Method Analysis of Drought Resistance in Rice at the Germination Stage

**DOI:** 10.3390/plants15203220

**Published:** 2026-10-20

**Authors:** Jiayu Song, Yutiao Chen, Bingyao Jiang, Linyou Wang

**Affiliations:** Institute of Crop and Nuclear Technology Utilization, Zhejiang Academy of Agricultural Sciences, Hangzhou 310000, China; songjy@zaas.ac.cn (J.S.); chenyutiao@sina.com (Y.C.)

**Keywords:** rice, drought, germination, membership function synthetic value, fisher discriminant, principal component analysis synthetic value, grading coefficient

## Abstract

Drought stress is a major abiotic factor limiting rice growth and productivity. Developing scientifically screened methods at the germination stage is critical for identifying superior drought-adapted genotypes. This study aimed to develop a novel discriminant equation for evaluating germination-stage drought resistance in rice, using field yield data as a benchmark to calibrate laboratory assessments. Seventy-six rice accessions from southern China were used as experimental materials. Drought resistance during germination was assessed using nine key phenotypic indicators. Three statistical methods—membership function synthetic value (MFSV), principal component analysis synthetic value (PCASV), and grading coefficient (GC)—were applied to classify drought resistance levels from different analytical perspectives. Field drought treatment was conducted throughout the growth period, and final grain yield was used to represent the comprehensive drought resistance (CDR)of each accession. Results showed only 45% of the accessions were consistently classified by all three statistical methods, indicating that the choice of analytical approach influences the outcome. In addition, MFSV, PCASV, and GC were all positively correlated with CDR, supporting the use of CDR as a reliable reference for integrating laboratory-based evaluations. Finally, a Fisher discriminant function was established using MFSV, PCASV, and GC as independent variables (X) and CDR as the dependent variable (Y), providing a more comprehensive and scientifically robust method for assessing drought resistance at the germination stage.

## 1. Introduction

Rice (*Oryza sativa* L.) is the staple food for more than half of the world’s population, and yield losses in rice pose a major threat to global food security [1]. Rice cultivation involves substantial water consumption, and irrigated rice represents 53% of the global cultivated rice area [2]. As global agricultural water resources dwindle, enhancing drought adaptability in rice has become pivotal for ensuring stable yields [3].

Rice is highly sensitive to drought stress and displays distinct morphological responses at different growth stages [4]. It is particularly susceptible during germination and maturity [5], which limits production and yield stability. The germination stage is highly sensitive to drought because it determines subsequent seedling growth and maturation. Therefore, screening rice cultivars for drought stress tolerance at the germination stage will provide new insights for rice breeding [6].

Large-scale screening of drought-tolerant rice germplasm is fundamental to elucidating the molecular mechanisms underlying drought resistance. In one study, 100 rice genotypes were evaluated for drought resistance at the germination and early seedling stages using the Standard Evaluation System, with germination index, seedling height, and dry weight as key criteria [7]. Similarly, Han et al. [8] evaluated drought resistance in 667 rice germplasm accessions using seedling root length under drought stress as the key indicator. They employed a genome-wide association study (GWAS) and identified a root-related gene designated as ROOT LENGTH 1 (*RoLe1*). Further investigation revealed that the OsNAC41-RoLe1-OsAGAP module promotes root development and drought resistance in upland rice. Additionally, the drought resistance of 99 rice accessions was evaluated under five distinct natural drought conditions, using mean trait values for statistical analysis. Results identified chlorophyll stability index, leaf rolling degree, days to 50% heading, chlorophyll content, root volume, and root biomass as optimal predictors of grain yield under stress. A subsequent GWAS based on these drought-resistant phenotypes revealed haplotype variations in genomic regions on chromosomes 1, 4, 6, and 11 [9]. In recent decades, a substantial number of quantitative trait loci have been identified using bi-parental or multi-parental populations [10,11,12]. GWAS and QTL mapping, based on population phenotyping, have become vital tools for deciphering the genetic basis of complex traits [13,14,15,16,17,18]. These studies highlight the importance of accurate phenotyping of large rice populations for mechanistic studies of drought resistance. However, drought susceptibility varies substantially with growth stage, drought duration, stress intensity, cultivar, and environment [19]. Variability in evaluation criteria and statistical methods across studies has led to inconsistent results and a lack of standardized protocols for assessment criteria, growth stages, and metrics [20]. This lack of standardization hinders progress in deciphering the mechanisms underlying drought resistance in rice. Consequently, there is an urgent need to integrate multi-trait screening approaches and develop scientifically robust predictive models.

Fisher discriminant analysis, also known as canonical discriminant analysis, identifies and establishes discriminant functions based on existing classification criteria and is widely applied in fields such as medicine, civil engineering, and agriculture [21,22,23]. The principle involves projecting high-dimensional independent variables into a low-dimensional discriminant space via linear transformation, maximizing inter-class separation while minimizing intra-class variability.

This study aimed to systematically evaluate drought resistance at the germination stage using multiple indicators and analytical methods, calibrate the germination data against field drought resistance, and thereby propose a novel assessment framework. Thus, this study selected 76 rice germplasm resources from southern China for evaluation under drought stress conditions during the germination stage. Nine key parameters were measured: germination potential (GP), germination rate (GR), bud index (BI), vitality index (VI), relative seedling water content (SRWC), germination drought resistance index (GDRI), root length (RL), shoot Length (SL), and root-to-shoot ratio (RSR). Furthermore, three statistical approaches, membership function synthetic value (MFSV), principal component analysis synthetic value (PCASV), and grading coefficient (GC) were employed to assess drought resistance during the germination stage of 76 rice accessions from multiple dimensions. Finally, Fisher’s discriminant function was established by designating the germination-stage drought resistance results derived from the three statistical methods as the independent variable (X) and the comprehensive drought resistance (CDR) of rice accessions as the dependent variable (Y), thereby proposing a novel and more scientific methodology for drought resistance evaluation. This study optimized the existing drought-resistance evaluation system for the rice germination stage and provided a theoretical foundation for drought-resistant rice breeding.

## 2. Results

### 2.1. Evaluation of Drought-Related Indicators During the Rice Germination Period

**Figure 1:**
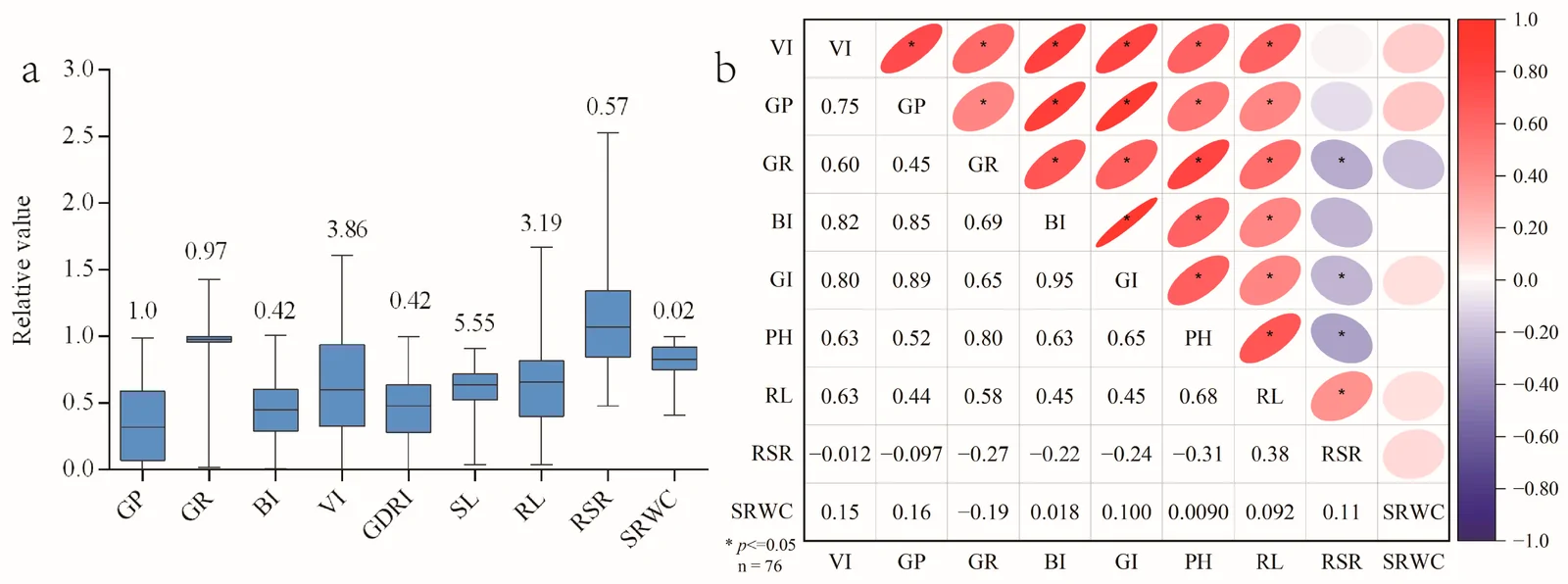
Box and whisker plots and correlation analysis of the relative values of the 9 indicators across 76 rice samples under drought stress. (**a**) The whisker below the box represents the first quartile or the fifth percentile showing the first 25% of the data distribution in this range. In contrast, the whisker above the box represents the third quartile or 95th percentile, showing the last 25% of the data distribution. The length of the box is called the interquartile range (25th to 75th percentile), showing 25% to 75% of the data distribution for that particular trait, and the horizontal line in the box indicates the median value. The number on each box represents the average value of the control. (**b**) Correlation analysis among the nine indicators. The lower-left values represent the correlation coefficients among the indicators; larger absolute values indicate stronger correlations, whereas negative values indicate inverse correlations. The asterisk (*) indicates significance, with *p* < 0.05 considered statistically significant. The color bar on the right indicates the magnitude of the correlation coefficients, and n denotes the sample size. The eccentricity of the ellipses reflects the strength of the correlations: the more elongated (narrower) the ellipse, the stronger the correlation. GP, germination potential; GR, germination rate; BI, bud index; VI, vitality index; SRWC, relative seedling water content; GDRI, germination drought resistance index; RL, root length; SL, shoot length; RSR, root-to-shoot ratio; MFSV, membership function synthetic value; PCASV, principal component analysis synthetic value; GC, grading coefficient.

The key indicators under drought stress were GP, GR, BI, VI, GDRI, SL, RL, RSR, and SRWC. The relative values of all indicators after drought stress and the average value of the control are shown in Figure 1a. GP was measured as the relative germination percentage on the fourth day of drought stress. A few highly drought-resistant rice materials exhibited GP values close to 100%, while drought-sensitive ones had GP values clustered in the 0–2% range. GR (relative germination percentage on day 8) approached 100% in most samples, indicating that drought stress delayed germination but had limited impact on the final GR. GI describes germination status from days 1 to 8, whereas GDRI applies decreasing weights over the same period to represent germination dynamics. GI and GDRI exhibited consistent trends (Figure 1). Nearly all materials reached their maximum germination rate by the sixth day, suggesting that an extended duration had minimal effects on germination. VI reflects both the number of germinated seeds and the growth status, as indicated by shoot Length and root length, under drought conditions. Higher VI values indicated stronger drought resistance during germination. For example, rice accession AS65 showed a GR of 1.0 and a relative VI of 1.61. RSR values ranged from 0.54 to 1.79, with about 50% of individuals exceeding 1.0. Comparisons between SL and RL revealed that drought stress generally reduced shoot Length, whereas root length increased in some cases, with a maximum RL value of 1.67. These results suggest that certain rice genotypes develop longer roots and higher root-to-shoot ratios under drought stress during the seedling stage. Coefficients of variation indicated that GP, VI, RL, and RSR were strongly influenced by drought stress, whereas GR and SRWC were less affected. Correlation analysis (Figure 1b) showed that RSR was not significantly correlated with VI, GP, or GI, and SRWC was not significantly correlated with any other indicator.

### 2.2. Multiple-Method Analysis of Comprehensive Drought Resistance in Rice

#### 2.2.1. MFSV

**Figure 2:**
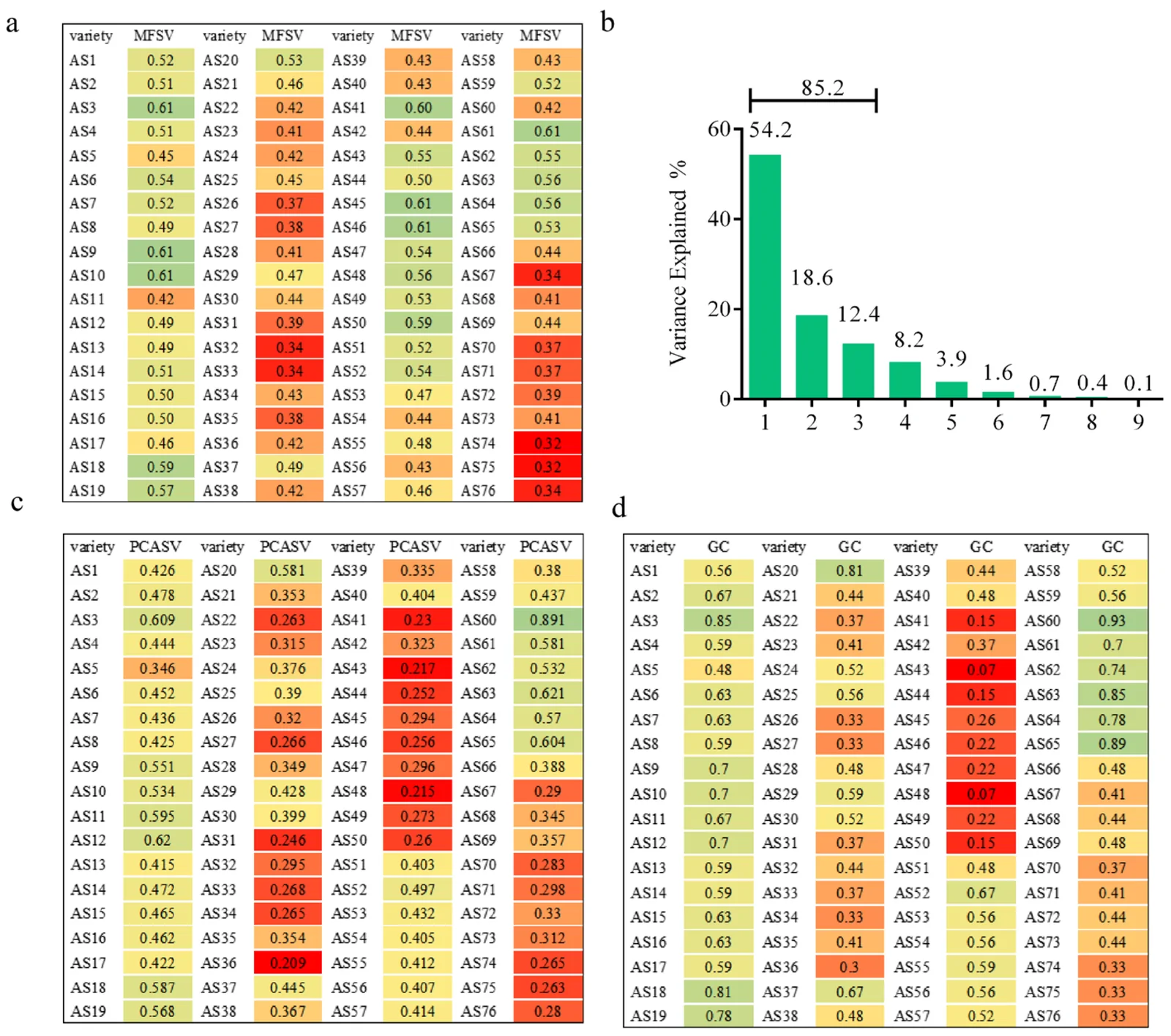
Evaluation of Drought Resistance in 76 Rice Accessions. (**a**) Drought resistance assessment based on MFSV (**membership function synthetic value**); (**b**) Principal component analysis of nine indicators; (**c**) Drought resistance evaluation based on PCASV (**principal component analysis synthetic value**); (**d**) Drought resistance assessment based on GC (**grading coefficient**). The numerical values in panels (**a**,**c**,**d**) indicate the drought resistance score of each accession. Higher scores represent greater drought resistance. The color scale ranges from green to red, with green indicating weak drought resistance and red indicating strong drought resistance.

The MFSV calculation incorporated the differential weighting of the nine indicators by computing the membership function value for each indicator, ultimately deriving the MFSV for each rice accession (Supplementary Table S2). Higher MFSV values indicated stronger drought resistance (Figure 2a). MFSV values ranged from 0.32 to 0.61, with most falling between 0.40 and 0.50, suggesting that most accessions had moderate drought resistance. Notably, AS9, AS10, AS45, and AS61 showed relatively strong drought resistance.

#### 2.2.2. PCASV

PCASV was obtained by performing PCA on the nine indicators, then calculating membership values for the principal components and combining them (Supplementary Table S3). PCA (Figure 2b) showed that the first three principal components accounted for 85.2% of the total variance, indicating that they effectively captured the variation in the original data. Higher PCASV values indicated stronger drought resistance. The PCASV values ranged from 0.21 to 0.62, with only a few rice materials exceeding 0.5, whereas the remaining materials exhibited a relatively uniform distribution (Figure 2c). Among the tested accessions, AS11, AS65, AS3, AS12, and AS63 exhibited the strongest drought resistance during germination. Notably, PCASV results differed somewhat from MFSV results.

#### 2.2.3. GC

GC scores were calculated by assigning weighted scores to each parameter based on its mean and standard deviation (Supplementary Table S4). Higher GC scores indicated greater drought resistance. Analysis of the GC scores identified AS18, AS20, AS3, AS63, and AS65 as exhibiting superior drought resistance during the germination phase (Figure 2d). Notably, GC results were partially inconsistent with MFSV and PCASV results.

#### 2.2.4. Consistency Analysis of MFSV, PCASV, and GC

The results from the three methods were separately ranked from weakest to strongest and then classified into four drought resistance levels (1 to 4). Each rice accession was assigned three separate “drought resistance level” identifications (A–C) (Table 1). These results were utilized for the subsequent Venn diagram analysis.

**Figure 3:**
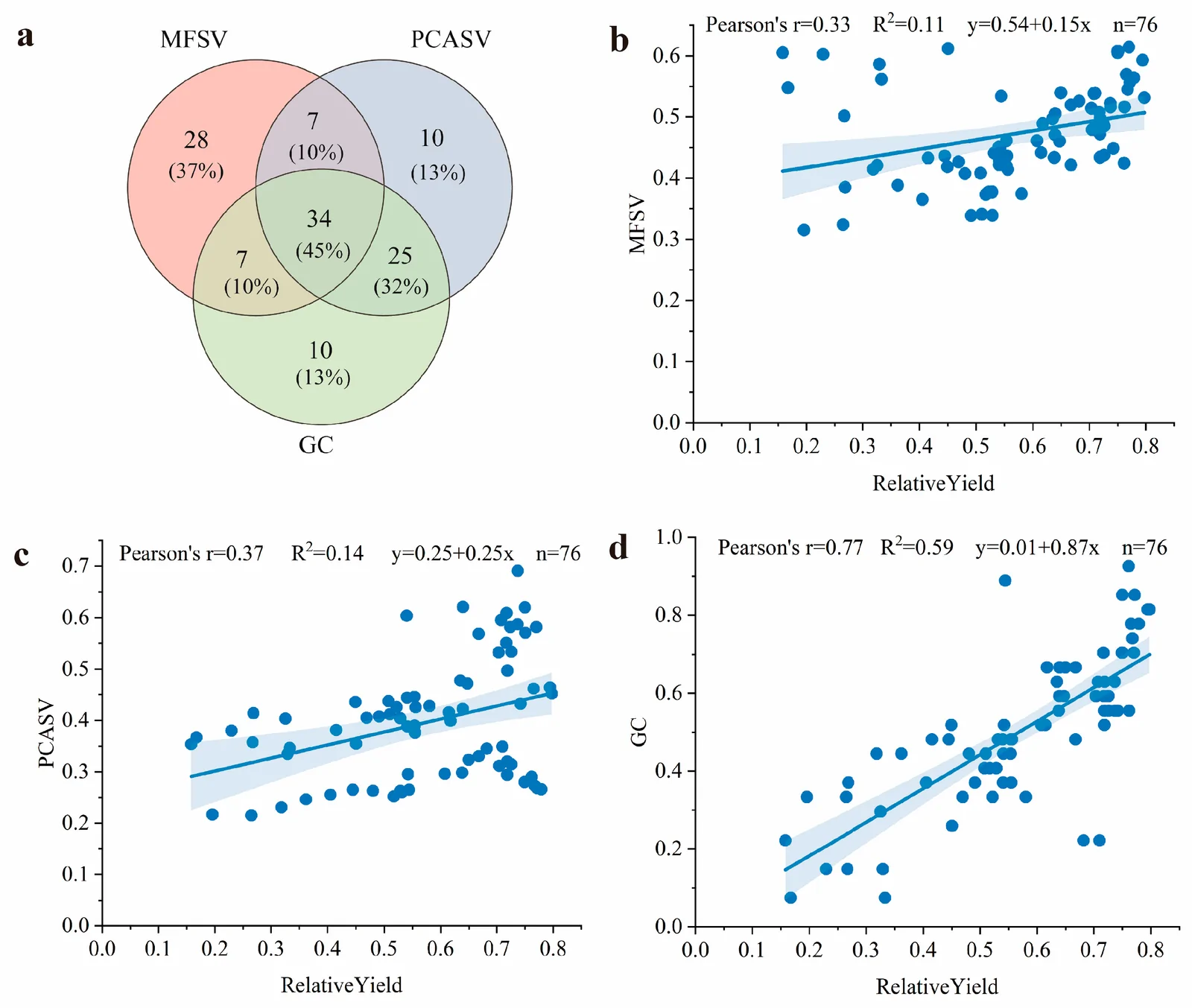
Integrated analysis of drought resistance classifications and their correlation with yield based on MFSV, PCASV, and GC methods. Note. (**a**) represents the Venn diagram of drought resistance results analyzed by MFSV, PCASV, and GC statistical methods. The different colors represent the three statistical methods (MFSV, PCASV, and GC). The values indicate the number of accessions within each section. Overlapping sections indicate accessions assigned the same drought resistance level by two or more statistical methods. Percentages represent the corresponding proportions of the total sample. (**b**–**d**) represent the Pearson correlation coefficients between different statistical methods and relative yield. Each point represents the value obtained using the corresponding method and relative yield value of a sample. The blue solid line represents the linear regression fit, and the light shaded area illustrates the concentration trend and variation range of the data points. MFSV, membership function synthetic value; PCASV, principal component analysis synthetic value; and GC, grading coefficient.

Venn diagram comparison of the drought resistance classifications derived from the three statistical methods (MFSV, PCASV, and GC; Figure 3a) revealed concordant classification of 45% of the accessions across all three methods. Agreement between MFSV and PCASV was 55%, between MFSV and GC 58%, and between GC and PCASV 79%. Overall, 97% of accessions showed consistent levels in at least two methods.

### 2.3. Assessment of Comprehensive Drought Resistance in Rice

Under drought conditions, yield served as the most direct indicator of rice drought resistance. All accessions were direct-seeded and grown in well-watered paddy fields and in moisture-deficient upland plots. The relative soil moisture content in the dryland plots was 10.7% of that in the control. After harvest, grain yield was measured, and the relative reduction rate of each sample was calculated (Supplementary Table S5). Under drought stress, accessions with different drought resistance levels showed distinct relative reduction rates. Based on these relative reduction rates, the rice population was classified into four drought resistance grades (Table 1).

Further correlation analysis between yield and values derived from the three statistical methods revealed positive relationships in all cases (Figure 3). Specifically, yield demonstrated a Pearson’s correlation coefficient of 0.77 with the GC, indicating a statistically significant positive correlation. These results confirm the reliability of yield as an indicator of CDR and provide a robust basis for the subsequent development of discriminant functions. To further verify this, the average germination drought resistance (AvgDGT) from the three methods and CDR were separately ranked. The Spearman rank correlation coefficient between the two sets of rankings was 0.76 (*p* < 0.01) (Supplementary Table S6). Under the conditions of this study, the drought resistance rankings of rice germplasm across growth stages showed a significant positive correlation.

### 2.4. Establishment of Fisher Discriminant Function

Fisher discriminant function equations were constructed using the MFSV, PCASV, and GC values as independent variables (X) (Table 1) and CDR as the dependent variable (Y) (Table 1). The Fisher discriminant function established four distinct categories: drought-sensitive, moderately drought-resistant, drought-resistant, and highly drought-resistant. The original re-substitution accuracy was 88.2%, whereas the leave-one-out cross-validation accuracy was 85.5%.

The Fisher discriminant functions were established as follows:

y = 257.64X_1_ + 6.4X_2_ + 232.96X_3_ + 168.71, for drought-sensitive

y = 209.74X_1_ + 13.21X_2_ + 181.1X_3_ + 110.09, for moderately drought-resistant

y = 169.44X_1_ + 9.64X_2_ + 135.5X_3_ + 67.04, for drought-resistant

y = 169.19X_1_ + 4.86X_2_ + 98.21X_3_ + 53.15, for highly drought-resistant

In these equations, X_1_, X_2_, and X_3_ represent MFSV, PCASV, and GC, respectively, and the constant term is the intercept. The drought resistance category was determined by selecting the equation that yielded the highest y-value.

## 3. Discussion

Screening for rice varieties with strong drought resistance is a prerequisite for research on both drought resistance mechanisms and breeding programs. Traditional field-based screening is highly susceptible to environmental interference, resulting in poor reproducibility, high labor intensity, and low efficiency. In contrast, laboratory-based screening at germination and seedling stages offers advantages such as simplicity, short cycles, and high throughput, enabling rapid identification of drought-tolerant germplasm [24,25]. The core determinants at the germination stage are seed water uptake, radicle protrusion, and dry matter accumulation, which are highly correlated with seed vigor and reserve mobilization. In contrast, drought resistance at the vegetative and reproductive stages primarily involves stomatal regulation, photosynthetic efficiency, photoassimilate allocation, and panicle development stability, all depending on overall stress tolerance and resource allocation. The physiological and molecular mechanisms of drought resistance differ markedly between germination and later stages. Therefore, predicting field drought resistance solely from germination-stage performance has often been questioned. However, because of its rapidity and simplicity, germination-stage assessment has also been used in some studies to inform later-stage evaluations [26,27]. To address this, we used yield measured after full-season drought treatment as a calibrating factor, establishing a calibration model based on a ‘laboratory screening-field validation’ framework. The calibrated model can predict plant drought resistance to a certain extent.

GP, GR, and BI reflect germination performance under drought, while SRWC, GDRI, and RSR help elucidate the impact of drought on seedling establishment after germination. VI integrates BI, SL, and RL, representing the single most informative indicator in germination-stage evaluation and thus a typical proxy for the germination process. Under drought stress, accessions with high VI can achieve rapid and uniform germination and maintain sufficient coleoptile and radicle elongation. This reflects differences in energy metabolism efficiency and the mobilization of stored reserves. In the present study, the drought-tolerant accession AS65 exhibited a GR of 1.0 and a relative VI of 1.61, which exemplifies this multiplicative effect of “efficiency and vigor.” GP reflects the uniformity and speed of germination within the first four days. Accessions with high GP are capable of rapid germination during the window period when moisture is still available, representing a manifestation of the drought-escape strategy. GP had the highest coefficient of variation among all indicators, indicating substantial genetic differentiation, and thus positioning GP as a highly discriminatory screening index [28,29,30]. Changes in shoot Length and root length occur when rice is subjected to drought during germination. In drought-resistant varieties, root length may not change or may even increase under drought stress. This response is attributed to the activation of inherent drought resistance mechanisms, where longer roots facilitate water uptake and improve drought resistance [31]. Among the materials tested, AS12 and AS20 showed reduced shoot Length but a significant increase in root length after drought treatment, resulting in a root-to-shoot ratio greater than 1. Therefore, these accessions are valuable for drought resistance research. GDRI is computed by cumulatively weighting daily germination percentages with decreasing coefficients, serving as a compressed representation of germination dynamics. Unlike GI, GDRI emphasizes early germination events, thus assessing seed response speed to osmotic stress from a temporal perspective. In this study, GDRI showed a trend similar to GI but with higher sensitivity, indicating that germination timing is an important drought-tolerance trait. SRWC and GDRI are specific indicators designed to assess drought resistance. However, our study revealed minimal variation in SRWC and no significant correlation with other metrics, and RSR also showed no significant correlation with VI, GP, or GI. We speculate that different rice varieties adopt distinct drought response strategies. Drought-avoiding types actively adapt by shortening their growth cycle to escape drought periods. Drought-tolerant types maintain homeostasis via deep roots, high water potential, and osmotic adjustment, which require sustained leaf water content. The low correlations and low/medium variations in SRWC and RSR observed in the current study population may be attributable to the coexistence of two types of strategies that offset each other’s trends. Therefore, these indicators may provide independent dimensions of drought resistance information, and such strategic diversity is biologically meaningful. Although SRWC showed no significant correlation with other indicators, its exclusion is not recommended. Many studies have evaluated drought resistance using limited indicators, often with membership function analysis [32,33].

MFSV, PCASV, and GC are three commonly used analytical methods in phenotypic statistics. Our results revealed inconsistencies between the three methods. Only 45% of accessions showed consistent resistance across all three methods, while 97% agreed in at least two. This suggests that using too many methods may introduce interference, which is why many studies rely on a single approach [34]. MFSV provides a dimension-free evaluation framework by standardizing indicators and integrating them with equal weights, suitable for initial comparisons when trait priorities are unknown. PCASV reduces correlated indicators into principal components to capture core variation patterns. It is valuable in exploratory research for revealing potential physiological mechanisms and trait associations when components have clear biological meaning. GC uses a non-parametric approach, converting continuous data into discrete ranks to assess relative performance within a population. Its insensitivity to absolute values makes it robust for large-scale germplasm screening and direct selection based on “superior/intermediate/poor” classifications. The 55% inconsistency for the same materials confirms that drought resistance is complex and not fully captured by any single method. This study avoids declaring a laboratory gold standard. Instead, it uses field-based grain yield under full-season drought as the definitive benchmark. By integrating the complementary information from the three methods via Fisher’s discriminant function, we built a theoretically sound and practically predictive model. Consequently, prior knowledge of the drought resistance attributes of the samples is required before constructing the discriminant function to ensure accurate positioning within the projected space. In this study, yield performance under field drought stress conditions was utilized as the criterion for assessing drought resistance in rice, primarily because yield serves as a direct manifestation of drought resistance and represents the most compelling evaluation metric.

This work demonstrates that drought resistance assessment should flexibly combine methods based on the objective and must always be validated against actual field performance. The correlations of MFSV (r = 0.33, R^2^ = 0.10) and PCASV (r = 0.37, R^2^ = 0.14) with CDR were relatively low, yet these r values indicate moderate positive correlations. Statistically, this indicates that germination-stage drought resistance can explain about 10–14% of the variation in field drought resistance. For complex quantitative traits, an indicator explaining >10% of phenotypic variation already has a reference value for breeding, as it exceeds random expectation. The GC index showed a strong correlation (r = 0.77, R^2^ = 0.59) and served as the core effective variable in Fisher’s discriminant function, with MFSV and PCASV as complementary dimensions to address the limitations of using GC alone. Our results also revealed a significant correlation (0.76) between the average drought resistance ranking at the germination stage and the CDR ranking, further supporting the feasibility of this approach. The GC method employs discrete grading based on the mean and standard deviation, thereby preserving the rank-order information of each indicator within the population. However, grain yield under drought stress is itself an integrated outcome arising from the balanced coordination of numerous physiological and morphological processes, and no single indicator can perfectly predict it. Consequently, indicators that maintain stable relative rankings within the population are more likely to approximate the underlying true performance, which also accounts for why GC exhibited the strongest correlation (r = 0.77) with field relative yield.

Admittedly, this method has certain limitations. The discriminant function model was developed using germplasm resources from southern China, with a relatively small sample size and insufficient genetic diversity. Future studies will expand the geographical origins and genetic diversity of the samples. In the present study, only a single PEG concentration was employed, and physiological evidence (e.g., electrolyte leakage, proline content, antioxidant enzyme activities) was not incorporated. Future work will apply different drought levels and include key physiological indicators to further refine the evaluation system. Furthermore, the discriminant model was validated only by internal leave-one-out cross-validation, without an external validation dataset. The results of this study can be applied to large-scale drought tolerance screening in rice, enabling variety selection to be matched to water availability. Drought-resistant and highly drought-resistant germplasm are recommended for cultivation in rainfed or water-limited regions to stabilize yield, whereas drought-sensitive germplasm should be restricted to irrigated paddy fields. The proposed method is simple and easy to implement, and allows drought resistance to be assessed at the early growth stage, thereby substantially shortening the evaluation cycle required for whole-growth-period drought resistance identification. Therefore, this approach is of considerable significance for rice breeding and production. However, as the model was constructed from 76 accessions of southern Chinese origin and validated only internally, recalibration with local check varieties is recommended prior to large-scale application.

## 4. Materials and Methods

### 4.1. Plant Materials and Growth Conditions

The experiment was conducted at the Institute of Crops and Nuclear Technology Utilisation, Zhejiang Academy of Agricultural Sciences, Hangzhou, Zhejiang, China. Seventy-six rice germplasm lines collected from southern China were used as experimental materials (Supplementary Table S1). All tested samples were harvested in October 2024 in Hangzhou, Zhejiang, China. Plump and uniformly sized seeds were selected from each accession and soaked in distilled water at 28 °C for 12 h. Subsequently, 50 seeds were evenly placed in sterile 90 mm-diameter Petri dishes lined with double-layer sterilized filter paper. For the drought stress treatment group, 15 mL of 20% polyethylene glycol (Macklin Biochemical Co., Ltd., Shanghai, China) solution was added to each dish, whereas the control group received an equal volume of distilled water [35,36,37,38]. Three replicates were performed for each treatment group. All dishes were maintained in a growth chamber (Zhejiang Top Cloud-Agri Technology Co., Ltd., Hangzhou, China) under controlled conditions of constant temperature (28 °C), relative humidity (70%), and a 10/14 h (light/dark) photoperiod for 10 d. The liquid level in each dish was monitored daily, and distilled water was replenished based on day one measurements to maintain a consistent volume throughout the experimental period.

### 4.2. Trait Measurement

All rice seeds were observed starting from the 2nd day of treatment. The germination standard was defined as the radicle breaking through the seed coat by 1 mm and the plumule reaching half the seed length. The number of germinated seeds was recorded at the same time on the 2nd, 4th, 6th, and 8th days. Germination potential was calculated on the 4th day, and the germination rate was calculated on the 8th day. On the 8th day, 15 plants were randomly selected from each Petri dish to measure root length and shoot Length. All samples in each Petri dish were individually weighed for fresh mass, then placed in an oven (Zhejiang Top Cloud-Agri Technology Co., Ltd., Hangzhou, China) at 105 °C for 0.5 h, followed by drying at 80 °C until a constant mass was reached, after which the dry mass was weighed.

The formulas for calculating germination potential, germination rate, germination index, vitality index, seedling water content, germination drought resistance index, shoot Length, root length, and root-to-shoot ratio are as follows:

GP = G4/50 × 100, where G4 is the number of germinated seeds on the 4th day, and 50 is the number of seeds per dish.

GR = G8/50 × 100, where G8 is the number of germinated seeds on the 8th day, and 50 is the number of seeds per dish.

BI = ∑(DG/DT), where DG is the number of daily germinated seeds, and DT is the corresponding germination time for DG.

VI = BI × (shoot Length + longest root length), where BI is the seed germination index.

SRWC = (seedling fresh mass − seedling dry mass)/seedling fresh mass × 100.

GI = (1.00) nd2 + (0.75) nd4 + (0.50) nd6 + (0.25) nd8, where nd2, nd4, nd6, and nd8 are the seed germination rates on the 2nd, 4th, 6th, and 8th days, respectively, and 1.00, 0.75, 0.50, and 0.25 are the weight coefficients assigned to the corresponding growth days.

GDRI = germination index of seeds under drought stress/germination index of seeds under control conditions.

RSR = root length/shoot Length.

RL = longest root length

SL = the height from the base of the plant root to the longest leaf

The data for all the above indicators are expressed as relative values:

Relative value of the indicator = measured value of the indicator under stress/measured value of the indicator under control conditions.

### 4.3. Calculation of MFSV, PCASV, and GC

The MFSV and PCASV were both calculated using the membership function method [39], while the GC value was obtained through the graded evaluation method [40]. The membership function value was calculated using the following formula:

(1) $$u\left(X_{j}\right)=\frac{X_{j}-X_{min}}{X_{max}-X_{min}}$$

where *j* = 1, 2, 3, …, *n*, *Xj* denotes the value of the *j*-th indicator; *X*min and *X*max represent the minimum and maximum values of the *j*-th indicator, respectively. The membership value *μ* for each indicator of each variety is computed accordingly.

The weight coefficient *W* is calculated as follows:

(2) $$W_{j}=r_{j}/\sum_{j=1}^{n}r_{j}$$

where *rj* denotes the coefficient of variation in the *j*-th indicator.

The comprehensive evaluation value *D* is then determined using:

(3) $$D=\sum_{j=1}^{n}(X_{j}\times W_{j})$$

where *j* = 1, 2, 3, …, *n*, *Xj* represents the membership value of the *j*-th indicator for each variety and *Wj* is the weight coefficient of the *j*-th indicator. A higher value of *D* indicates a better performance of the trait.

MFSV was calculated using the membership function above, with j = 1–9 corresponding to GP, GR, SRWC, GI, VI, GDRI, SL, RL, and RSR. The MFSV is the sum of Xj × Wj for the nine indicators of each rice variety. Higher MFSV values indicate stronger drought resistance, whereas lower values indicate weaker drought resistance.

The PCASV was obtained by performing principal component analysis on the nine indicators and then applying the aforementioned membership function calculation formula. Three principal components were extracted. The weights Wj (j = 1–3) were based on the contribution rates of PC1, PC2, and PC3. PCASV is the sum of Xj × Wj for the three principal components for each variety. Higher PCASV values indicate stronger drought resistance, whereas lower values indicate weaker drought resistance.

GC Calculation Method: The mean value ($\bar{Xj}$)$\bar{Xj}$ of each of the nine indicators was used as the benchmark, and the standard deviation (*S*) was used as the criterion for grade classification. Each indicator value *Xj* was assigned a grade as follows:

Grade 1: *Xj* ≥ $\bar{Xj}$+ *S*, Grade 2: $\bar{Xj}$+ *S* > *Xj* ≥ $\bar{Xj}$, Grade 3: $\bar{Xj}$ > *Xj* ≥ $\bar{Xj}$− *S*, Grade 4: $\bar{Xj}$− *S* > *Xj*.

GC was calculated as follows:

GC = (*nm* − ∑*Xj*)/(*nm* − *n*) (4)

where *n* is the number of indicators, *m* is the number of grades, and *Xj* denotes the graded evaluation value of the *j*-th indicator. The sum of *Xj* values represents the drought resistance grade value of each sample. A larger GC value indicates stronger drought resistance in the variety.

### 4.4. Evaluation of Rice Drought Resistance at the Germination Stage

The Fisher discriminant function takes MFSV, PCASV, and GC as independent variables and “drought resistance level” as the dependent variable. In this study, the method for obtaining the “drought resistance level” of rice was as follows: the results of the three groups (MFSV, PCASV, and GC) were sorted from weak to strong. The top 15% of the total number after sorting were classified as level 1, 16–50% as level 2, 51–85% as level 3, and 86–100% as level 4 for the “drought resistance level” division [41].

### 4.5. Assessment of CDR in Rice

For each variety, 400 plump and uniform seeds were selected. After 1 day of soaking, the seeds were direct-seeded. The control group was established in conventional paddy fields, while the drought treatment group was planted in relatively moist upland soil. The plot size for both conditions was 1 × 10 square meters, and an equal amount of base fertilizer was applied to all plots before sowing. The water management protocol for the drought treatment group was as follows: after sowing, a small amount of water was sprayed every 5 days for a total of 2–3 times until seedling emergence, after which water supply was stopped. During the heading stage, plots were irrigated every 4 days for a total of 3–4 times. No additional manual irrigation was provided at other times.

Soil moisture content (SMC) was determined gravimetrically [42]. Briefly, approximately 1 kg of fresh soil was weighed to obtain its wet weight, then oven-dried at 105 °C until constant weight to determine the dry weight. SMC was calculated as:

SMC (%) = [(wet weight − dry weight)/dry weight] × 100

Under drought treatment, SMC was measured at the seedling (S1), booting (S2), and maturity (S3) stages, and recorded as TSMC1, TSMC2, and TSMC3, respectively. For the control (CK), corresponding SMC values from conventionally irrigated paddy fields at the same growth stages were recorded as CKSMC1, CKSMC2, and CKSMC3.

The average relative soil moisture content under drought treatment was then calculated as: Average relative SMC = [(TSMC1/CKSMC1) + (TSMC2/CKSMC2) + (TSMC3/CKSMC3)]/3At maturity, all plants from both the control and treatment groups were harvested and weighed. The Reduction rate for each sample was calculated (Reduction rate = (Control Group − Drought Treatment Group)/Control Group) and ranked from highest to lowest. The top 15% of the total number after sorting were classified as level 1, 16–50% as level 2, 51–85% as level 3, and 86–100% as level 4 for the “CDR”.

### 4.6. Fisher Discriminant

Leave-one-out cross-validation (LOOCV) was performed using SPSS 26. The independent variables (predictors) included MFSV, PCASV, and GC, while the dependent variable (outcome) was the comprehensive drought resistance rating (CDR) determined by field yield. The grouping variable was defined with a custom range of 1–4. In the Classify sub-dialog, Summary table and Leave-one-out classification were retained; in the Save sub-dialog, Predicted group membership and Probabilities of group membership were selected. Fisher discriminant functions were constructed as follows [21,22,23]:

y = u_1_X_1_ + u_2_X_2_ + u_3_X_3_ + P

where X_1_, X_2_, and X_3_ represent the drought resistance ratings based on MFSV, PCASV, and GC, respectively; u_1_, u_2_, and u_3_ denote the coefficients corresponding to X_1_, X_2_, and X_3_; P is the constant term; and y indicates the level of drought resistance. In this study, four discriminant functions were established: y_1_ for highly drought-resistant, y_2_ for drought-resistant, y_3_ for moderately drought-resistant, and y_4_ for drought-sensitive. The total sample size should be greater than the sum of the number of features and the number of classes (3 + 4 = 7), and a robust recommendation is that the sample size per class should be at least 3–6 times the number of features (9–18) [43]. In this study, the total sample size is 76, with an average of 19 samples per class. Therefore, the model is reasonably reliable, and the cross-validation results are stable.

### 4.7. Statistical Analysis

Data were processed using Microsoft Office 2010 software (Microsoft Corp., Redmond, WA, USA). All experimental treatments were conducted in triplicate. Statistical significance and error analysis were performed using SPSS 26 (IBM Corp., Armonk, NY, USA), with significant differences defined at * *p* ≤ 0.05 according to Tukey’s test. The membership function values were calculated using SPSS 26. Significant difference diagrams were generated using Origin 2022 (OriginLab Corp., Northampton, MA, USA), and box plots were created using SigmaPlot 12.5 (Systat Software Inc., San Jose, CA, USA).

## 5. Conclusions

Drought stress is a highly destructive abiotic factor that limits rice growth and development. Establishing an efficient and rapid screening technique during the germination stage is crucial for identifying genotypes that are best suited to limited water conditions. Traits such as GP, GR, BI, VI, GDRI, SL, RL, RSR, and SRWC reflect various aspects of rice growth from germination to early seedling establishment under drought stress. Drought resistance evaluations derived from the MFSV, PCASV, and GC statistical methods showed discrepancies, with only 45% of the accessions exhibiting consistent results across all three methods. Therefore, screening for drought-resistant germplasm should incorporate multiple statistical approaches. Based on the results of these three methods, and using CDR as a calibration reference factor, we propose a new discriminant equation approach for assessing drought resistance levels during rice germination. The drought-resistant genotypes identified in this study can be used to explore the mechanisms underlying drought resistance, which are essential for achieving and maintaining global food security.

## Figures and Tables

**Table 1:** Predicted drought resistance classifications of 76 rice varieties.

No.	A	B	C	CDR	No.	A	B	C	CDR	No.	A	B	C	CDR
AS1	2	2	2	2	AS27	4	3	3	3	AS53	2	2	2	2
AS2	2	2	2	2	AS28	3	3	3	3	AS54	3	2	2	2
AS3	1	1	1	1	AS29	2	2	2	2	AS55	2	2	2	2
AS4	2	2	2	2	AS30	3	2	2	2	AS56	3	2	2	2
AS5	3	3	3	3	AS31	4	4	3	4	AS57	3	2	2	2
AS6	2	2	2	2	AS32	4	3	3	3	AS58	3	3	3	3
AS7	2	2	2	2	AS33	4	3	3	3	AS59	2	2	2	2
AS8	2	2	2	2	AS34	3	4	3	3	AS60	3	1	1	1
AS9	1	2	1	1	AS35	4	3	3	3	AS61	1	1	2	1
AS10	1	2	1	1	AS36	3	4	4	4	AS62	2	2	1	2
AS11	3	1	2	2	AS37	2	2	2	2	AS63	2	1	1	1
AS12	2	1	2	2	AS38	3	3	3	3	AS64	1	1	1	1
AS13	2	2	2	2	AS39	3	3	3	3	AS65	2	1	1	1
AS14	2	2	2	2	AS40	3	2	3	3	AS66	3	3	3	3
AS15	2	2	2	2	AS41	1	4	4	4	AS67	4	3	3	3
AS16	2	2	2	2	AS42	3	3	3	3	AS68	3	3	3	3
AS17	3	2	2	2	AS43	2	4	4	4	AS69	3	3	3	3
AS18	1	1	1	1	AS44	2	4	4	4	AS70	4	3	3	3
AS19	1	1	1	1	AS45	1	3	4	3	AS71	4	3	3	3
AS20	2	1	1	1	AS46	1	4	4	4	AS72	3	3	3	3
AS21	3	3	3	3	AS47	2	3	4	2	AS73	3	3	3	3
AS22	3	4	3	3	AS48	2	4	4	4	AS74	4	4	3	4
AS23	3	3	3	3	AS49	2	3	4	2	AS75	4	4	4	4
AS24	3	3	2	3	AS50	1	4	4	4	AS76	4	3	4	4
AS25	3	2	2	2	AS51	2	2	3	2					
AS26	4	3	3	3	AS52	2	2	2	2					

Note: A, B, and C represent the drought resistance levels based on MFSV, PCASV, and GC, respectively; i.e., the results of the three groups were sorted from weak to strong. The top 15% of the total number after sorting were classified as level 1, 16–50% as level 2, 51–85% as level 3, and 86–100% as level 4. CDR represents comprehensive drought resistance, i.e., all tested samples were ranked in descending order of reduction rate, and the top 15% of the total number after sorting were classified as level 1, 16–50% as level 2, 51–85% as level 3, and 86–100% as level 4 for the “CDR”.

## Data Availability

The data are contained within the manuscript. All data, tables, and figures in the manuscript are original.

## Supplementary Materials

The following supporting information can be downloaded at: https://www.mdpi.com/article/10.3390/plants15203220/s1, Supplementary Table S1: Information on 76 rice accessions; Supplementary Table S2: Statistical analysis of drought resistance during germination stage using MFSV; Supplementary Table S3: Statistical analysis of drought resistance during germination stage using PCASV; Supplementary Table S4: Statistical analysis of drought resistance during germination stage using GC; Supplementary Table S5: Drought resistance classification and reduction rate performance of rice; Supplementary Table S6: Spearman’s rank correlation coefficient calculated from the AvgDGT and CDR.

## Abbreviations

MFSV	Membership Function Synthetic Value
PCASV	Principal Component Analysis Synthetic Value
GC	Grading Coefficient
GP	Germination Potential
GR	Germination Rate
BI	Bud Index
VI	Vitality Index
SRWC	Relative Seedling Water Content
GDRI	Germination Drought Resistance Index
RL	Root Length
SL	Shoot Length
RSR	Root-To-Shoot Ratio
CDR	Comprehensive Drought Resistance
