# Supplementary material for: Phenotypic Variation Characterization and Multiple-Method Analysis of Drought Resistance in Rice at the Germination Stage
Source: Plants (Basel). 2026 Oct 20;15(20):3220. doi: 10.3390/plants15203220 (PMC13708856; doi:10.3390/plants15203220)
Supplement: Supplementary file 1 [file plants-15-03220-s001.zip › Supplementary Table S3.pdf]

Supplementary Table S3. Statistical analysis of drought resistance during germination stage using PCASV

NO.	Membership Values of Principal Components			PCASV of Principal Components			PCASV	Ranking
AS1	0.453	0.176	0.685	0.288	0.039	0.099	0.426	27
AS2	0.482	0.263	0.784	0.307	0.058	0.114	0.478	16
AS3	0.598	0.610	0.656	0.380	0.133	0.095	0.609	4
AS4	0.507	0.151	0.609	0.323	0.033	0.088	0.444	22
AS5	0.443	0.036	0.388	0.282	0.008	0.056	0.346	47
AS6	0.487	0.180	0.709	0.310	0.039	0.103	0.452	20
AS7	0.468	0.227	0.610	0.298	0.050	0.088	0.436	24
AS8	0.468	0.154	0.646	0.298	0.034	0.094	0.425	28
AS9	0.641	0.157	0.750	0.408	0.034	0.109	0.551	12
AS10	0.607	0.164	0.768	0.386	0.036	0.111	0.534	13
AS11	0.580	0.957	0.116	0.369	0.209	0.017	0.595	6
AS12	0.594	1.000	0.159	0.378	0.219	0.023	0.620	3
AS13	0.424	0.292	0.566	0.270	0.064	0.082	0.415	30
AS14	0.519	0.147	0.754	0.330	0.032	0.109	0.472	17
AS15	0.499	0.359	0.475	0.317	0.079	0.069	0.465	18
AS16	0.482	0.334	0.566	0.307	0.073	0.082	0.462	19
AS17	0.447	0.334	0.449	0.284	0.073	0.065	0.422	29
AS18	0.659	0.297	0.708	0.419	0.065	0.103	0.587	7
AS19	0.617	0.312	0.742	0.393	0.068	0.108	0.568	11
AS20	0.649	0.339	0.651	0.413	0.074	0.094	0.581	8
AS21	0.408	0.189	0.361	0.260	0.041	0.052	0.353	45
AS22	0.372	0.079	0.063	0.237	0.017	0.009	0.263	67
AS23	0.328	0.194	0.437	0.209	0.042	0.063	0.315	53
AS24	0.451	0.135	0.413	0.287	0.030	0.060	0.376	41
AS25	0.451	0.173	0.448	0.287	0.038	0.065	0.390	38
AS26	0.366	0.181	0.328	0.233	0.040	0.047	0.320	52
AS27	0.330	0.151	0.158	0.210	0.033	0.023	0.266	64
AS28	0.365	0.310	0.338	0.232	0.068	0.049	0.349	46
AS29	0.473	0.302	0.416	0.301	0.066	0.060	0.428	26
AS30	0.455	0.186	0.474	0.290	0.041	0.069	0.399	37
AS31	0.273	0.253	0.120	0.173	0.055	0.017	0.246	72
AS32	0.270	0.392	0.260	0.172	0.086	0.038	0.295	57
AS33	0.281	0.295	0.170	0.179	0.065	0.025	0.268	63
AS34	0.341	0.134	0.132	0.217	0.029	0.019	0.265	65
AS35	0.356	0.290	0.444	0.227	0.063	0.064	0.354	44

AS36	0.243	0.251	0.000	0.155	0.055	0.000	0.209	76
AS37	0.483	0.267	0.553	0.307	0.058	0.080	0.445	21
AS38	0.348	0.281	0.577	0.221	0.061	0.084	0.367	42
AS39	0.424	0.000	0.448	0.270	0.000	0.065	0.335	49
AS40	0.367	0.301	0.721	0.234	0.066	0.105	0.404	35
AS41	0.036	0.392	0.838	0.023	0.086	0.122	0.230	73
AS42	0.262	0.273	0.668	0.167	0.060	0.097	0.323	51
AS43	0.055	0.343	0.735	0.035	0.075	0.107	0.217	74
AS44	0.117	0.266	0.823	0.075	0.058	0.119	0.252	71
AS45	0.091	0.587	0.743	0.058	0.128	0.108	0.294	58
AS46	0.000	0.561	0.916	0.000	0.123	0.133	0.256	70
AS47	0.127	0.459	0.793	0.081	0.100	0.115	0.296	56
AS48	0.009	0.321	0.958	0.005	0.070	0.139	0.215	75
AS49	0.060	0.588	0.730	0.038	0.129	0.106	0.273	62
AS50	0.056	0.395	0.955	0.035	0.086	0.138	0.260	69
AS51	0.455	0.121	0.602	0.289	0.026	0.087	0.403	36
AS52	0.557	0.122	0.800	0.354	0.027	0.116	0.497	15
AS53	0.502	0.171	0.516	0.320	0.038	0.075	0.432	25
AS54	0.473	0.106	0.557	0.301	0.023	0.081	0.405	34
AS55	0.503	0.041	0.570	0.320	0.009	0.083	0.412	32
AS56	0.465	0.232	0.417	0.296	0.051	0.060	0.407	33
AS57	0.478	0.047	0.687	0.304	0.010	0.100	0.414	31
AS58	0.458	0.054	0.530	0.291	0.012	0.077	0.380	40
AS59	0.518	0.053	0.663	0.330	0.012	0.096	0.437	23
AS60	1.000	0.801	0.546	0.636	0.175	0.079	0.891	1
AS61	0.630	0.162	1.000	0.401	0.035	0.145	0.581	8
AS62	0.569	0.213	0.853	0.362	0.047	0.124	0.532	14
AS63	0.695	0.260	0.839	0.442	0.057	0.122	0.621	2
AS64	0.599	0.278	0.884	0.381	0.061	0.128	0.570	10
AS65	0.645	0.313	0.859	0.411	0.069	0.125	0.604	5
AS66	0.349	0.194	0.851	0.222	0.042	0.123	0.388	39
AS67	0.273	0.169	0.548	0.174	0.037	0.079	0.290	59
AS68	0.338	0.219	0.568	0.215	0.048	0.082	0.345	48
AS69	0.314	0.306	0.625	0.200	0.067	0.091	0.357	43
AS70	0.303	0.152	0.395	0.193	0.033	0.057	0.283	60
AS71	0.294	0.205	0.459	0.187	0.045	0.066	0.298	55
AS72	0.337	0.203	0.492	0.214	0.044	0.071	0.330	50
AS73	0.354	0.173	0.333	0.225	0.038	0.048	0.312	54
AS74	0.278	0.108	0.447	0.177	0.024	0.065	0.265	65
AS75	0.316	0.054	0.347	0.201	0.012	0.050	0.263	67
AS76	0.281	0.145	0.480	0.179	0.032	0.070	0.280	61

weight coefficient	0.636	0.219	0.145

PCASV represents principal component analysis synthetic value
