# Supplementary material for: Phenotypic Variation Characterization and Multiple-Method Analysis of Drought Resistance in Rice at the Germination Stage
Source: Plants (Basel). 2026 Oct 20;15(20):3220. doi: 10.3390/plants15203220 (PMC13708856; doi:10.3390/plants15203220)
Supplement: Supplementary file 1 [file plants-15-03220-s001.zip › Supplementary Table S2.pdf]

Supplementary Table S2. Statistical analysis of drought resistance during germination stage using MFSV

NO.	MFSV of each indicator									MFSV	Ranking
	GP	GR	BI	VI	GDRI	PH	RL	RSR	SRWC		
AS1	0.08	0.028	0.022	0.098	0.027	0.002	0.071	0.017	0.17	0.516	24
AS2	0.051	0.03	0.015	0.087	0.034	0.002	0.067	0.026	0.194	0.505	28
AS3	0.078	0.037	0.017	0.123	0.03	0.001	0.096	0.06	0.162	0.605	5
AS4	0.097	0.032	0.024	0.105	0.021	0.002	0.061	0.015	0.151	0.507	27
AS5	0.118	0.028	0.031	0.112	0.006	0.002	0.054	0.004	0.096	0.451	42
AS6	0.076	0.03	0.022	0.12	0.025	0.002	0.07	0.018	0.176	0.538	18
AS7	0.069	0.029	0.024	0.098	0.047	0.002	0.08	0.022	0.151	0.522	22
AS8	0.061	0.029	0.021	0.097	0.044	0.002	0.062	0.015	0.16	0.491	32
AS9	0.119	0.04	0.028	0.14	0.021	0	0.065	0.015	0.186	0.614	1
AS10	0.095	0.038	0.026	0.148	0.027	0.001	0.066	0.016	0.19	0.607	3
AS11	0.092	0.036	0.02	0.022	0.006	0.003	0.119	0.094	0.029	0.421	56
AS12	0.125	0.037	0	0.093	0.065	0.003	0.026	0.098	0.039	0.488	34
AS13	0.068	0.026	0.018	0.105	0.021	0.002	0.076	0.029	0.14	0.485	35
AS14	0.066	0.032	0.02	0.08	0.056	0.002	0.057	0.014	0.187	0.514	26
AS15	0.05	0.031	0.021	0.092	0.06	0.002	0.089	0.035	0.118	0.498	30
AS16	0.057	0.03	0.02	0.078	0.056	0.002	0.08	0.033	0.14	0.497	31
AS17	0.071	0.028	0.02	0.072	0.048	0.003	0.075	0.033	0.111	0.461	39
AS18	0.12	0.041	0.027	0.112	0.035	0	0.053	0.029	0.175	0.593	8
AS19	0.088	0.039	0.023	0.116	0.036	0	0.054	0.031	0.184	0.569	10
AS20	0.117	0.041	0.024	0.078	0.041	0.001	0.035	0.033	0.161	0.531	20
AS21	0.055	0.025	0.027	0.104	0.06	0.002	0.078	0.019	0.089	0.461	39
AS22	0.072	0.023	0.037	0.124	0.067	0.003	0.072	0.008	0.016	0.422	55

AS23	0.054	0.02	0.022	0.08	0.039	0.002	0.062	0.019	0.108	0.408	62
AS24	0.062	0.028	0.027	0.024	0.102	0.003	0.056	0.013	0.102	0.418	59
AS25	0.075	0.028	0.025	0.084	0.047	0.002	0.061	0.017	0.111	0.449	43
AS26	0.091	0.023	0.023	0.081	0.013	0.002	0.043	0.018	0.081	0.374	68
AS27	0.093	0.021	0.031	0.089	0.033	0.003	0.053	0.015	0.039	0.377	66
AS28	0.046	0.023	0.018	0.077	0.046	0.003	0.088	0.03	0.084	0.414	60
AS29	0.064	0.03	0.023	0.089	0.051	0.003	0.079	0.03	0.103	0.471	38
AS30	0.075	0.028	0.023	0.07	0.049	0.003	0.058	0.018	0.117	0.442	44
AS31	0.098	0.017	0.028	0.106	0.022	0.003	0.057	0.025	0.03	0.385	65
AS32	0.074	0.017	0.011	0.085	0.012	0.003	0.038	0.039	0.064	0.341	71
AS33	0.093	0.018	0.02	0.076	0.022	0.003	0.037	0.029	0.042	0.339	72
AS34	0.098	0.021	0.036	0.091	0.053	0.003	0.077	0.013	0.033	0.427	53
AS35	0.09	0.022	0.014	0.042	0.022	0.002	0.047	0.029	0.11	0.377	66
AS36	0.133	0.015	0.036	0.089	0.05	0.004	0.069	0.025	0	0.421	56
AS37	0.059	0.03	0.021	0.091	0.05	0.002	0.073	0.026	0.137	0.489	33
AS38	0.032	0.022	0.013	0.091	0.024	0.002	0.065	0.028	0.143	0.419	58
AS39	0.095	0.026	0.029	0.088	0.03	0.002	0.047	0	0.111	0.429	52
AS40	0.049	0.023	0.01	0.064	0.017	0.001	0.06	0.03	0.179	0.433	51
AS41	0.086	0.002	0.042	0.094	0.05	0.003	0.08	0.039	0.208	0.603	7
AS42	0.095	0.016	0.016	0.016	0.027	0.002	0.072	0.027	0.165	0.436	47
AS43	0.097	0.003	0.041	0.076	0.042	0.003	0.07	0.034	0.182	0.548	14
AS44	0.081	0.007	0.034	0.042	0.026	0.002	0.08	0.026	0.204	0.502	29
AS45	0.095	0.006	0.019	0.102	0.073	0.003	0.073	0.058	0.184	0.612	2
AS46	0.093	0	0.03	0.112	0.06	0.003	0.024	0.055	0.227	0.605	5
AS47	0.091	0.008	0.02	0.06	0.05	0.002	0.067	0.045	0.196	0.539	17
AS48	0.077	0.001	0.048	0.078	0.02	0.002	0.067	0.032	0.237	0.562	12

AS49	0.103	0.004	0.015	0.103	0.059	0.003	0	0.058	0.181	0.526	21
AS50	0.079	0.003	0.036	0.072	0.038	0.002	0.08	0.039	0.237	0.586	9
AS51	0.109	0.028	0.027	0.081	0.026	0.003	0.084	0.012	0.149	0.52	23
AS52	0.049	0.035	0.021	0.075	0.092	0.002	0.054	0.012	0.198	0.54	16
AS53	0.099	0.031	0.024	0.071	0.039	0.003	0.06	0.017	0.128	0.472	37
AS54	0.099	0.03	0.022	0.029	0.045	0.003	0.062	0.01	0.138	0.437	46
AS55	0.113	0.031	0.026	0.048	0.041	0.002	0.073	0.004	0.141	0.479	36
AS56	0.113	0.029	0.022	0.039	0.043	0.003	0.058	0.023	0.103	0.434	49
AS57	0.095	0.03	0.021	0.019	0.044	0.002	0.075	0.005	0.17	0.461	39
AS58	0.09	0.029	0.025	0.037	0.054	0.003	0.061	0.005	0.131	0.434	49
AS59	0.096	0.032	0.026	0.059	0.059	0.002	0.073	0.005	0.164	0.516	24
AS60	0	0.062	0.077	0.035	0.011	0.003	0.022	0.079	0.135	0.424	54
AS61	0.095	0.039	0.018	0.121	0.006	0.009	0.055	0.016	0.248	0.607	3
AS62	0.058	0.036	0.017	0.098	0.038	0.012	0.055	0.021	0.211	0.545	15
AS63	0.085	0.043	0.025	0.067	0.08	0.001	0.021	0.026	0.208	0.556	13
AS64	0.083	0.037	0.018	0.07	0.044	0.004	0.061	0.027	0.219	0.564	11
AS65	0.101	0.04	0.017	0.033	0.056	0	0.042	0.031	0.213	0.534	19
AS66	0.096	0.022	0.011	0	0	0.003	0.074	0.019	0.211	0.436	47
AS67	0.058	0.017	0.018	0.064	0.004	0.002	0.024	0.017	0.136	0.339	72
AS68	0.029	0.021	0.015	0.086	0.033	0.002	0.06	0.022	0.141	0.407	63
AS69	0.014	0.02	0.011	0.09	0.032	0.002	0.088	0.03	0.155	0.441	45
AS70	0.036	0.019	0.021	0.091	0.036	0.002	0.047	0.015	0.098	0.365	70
AS71	0.036	0.018	0.018	0.084	0.03	0.002	0.051	0.02	0.114	0.373	69
AS72	0.037	0.021	0.017	0.08	0.035	0.002	0.053	0.02	0.122	0.388	64
AS73	0.035	0.022	0.024	0.102	0.058	0.003	0.071	0.017	0.082	0.414	60
AS74	0.051	0.017	0.022	0.062	0.023	0.002	0.018	0.011	0.111	0.315	76

AS75	0.057	0.02	0.025	0.066	0.041	0.003	0.021	0.005	0.086	0.324	75
AS76	0.044	0.018	0.02	0.07	0.022	0.002	0.029	0.014	0.119	0.338	74
weight coefficient	0.133	0.062	0.077	0.148	0.102	0.012	0.119	0.098	0.248		

GP represents germination potential, GRrepresents germination rate, BI represents bud index, VI represents vitality index, SRWC represents seedling relative water content, GDRI represents germination drought resistance index, RL represents root length, PH represents plant height, RSR represents root-to-shoot ratio, MFSV represents membership function synthetic value.
