# Supplementary material for: Phenotypic Variation Characterization and Multiple-Method Analysis of Drought Resistance in Rice at the Germination Stage
Source: Plants (Basel). 2026 Oct 20;15(20):3220. doi: 10.3390/plants15203220 (PMC13708856; doi:10.3390/plants15203220)
Supplement: Supplementary file 1 [file plants-15-03220-s001.zip › Supplementary Table S1.pdf]

Supplementary Table S1. Information on 76 rice accessions

NO.	Category	NO.	Category	NO.	Category	NO.	Category
AS1	Indica-leaning rice	AS20	Japonica-leaning rice	AS39	Japonica-leaning rice	AS58	Indica-leaning rice
AS2	Indica-leaning rice	AS21	Japonica-leaning rice	AS40	Japonica-leaning rice	AS59	Indica-leaning rice
AS3	Indica-leaning rice	AS22	Japonica-leaning rice	AS41	Japonica-leaning rice	AS60	Japonica-leaning rice
AS4	Indica-leaning rice	AS23	Japonica-leaning rice	AS42	Japonica-leaning rice	AS61	Japonica-leaning rice
AS5	Indica-leaning rice	AS24	Japonica-leaning rice	AS43	Japonica-leaning rice	AS62	Indica-leaning rice
AS6	Indica-leaning rice	AS25	Japonica-leaning rice	AS44	Japonica-leaning rice	AS63	Japonica-leaning rice
AS7	Indica-leaning rice	AS26	Japonica-leaning rice	AS45	Indica-leaning rice	AS64	Japonica-leaning rice
AS8	Indica-leaning rice	AS27	Indica-leaning rice	AS46	Indica-leaning rice	AS65	Japonica-leaning rice
AS9	Indica-leaning rice	AS28	Indica-leaning rice	AS47	Indica-leaning rice	AS66	Japonica-leaning rice
AS10	Japonica-leaning rice	AS29	Indica-leaning rice	AS48	Indica-leaning rice	AS67	Indica-leaning rice
AS11	Japonica-leaning rice	AS30	Indica-leaning rice	AS49	Indica-leaning rice	AS68	Indica-leaning rice
AS12	Indica-leaning rice	AS31	Indica-leaning rice	AS50	Indica-leaning rice	AS69	Indica-leaning rice
AS13	Indica-leaning rice	AS32	Indica-leaning rice	AS51	Japonica-leaning rice	AS70	Indica-leaning rice
AS14	Indica-leaning rice	AS33	Indica-leaning rice	AS52	Japonica-leaning rice	AS71	Indica-leaning rice
AS15	Indica-leaning rice	AS34	Indica-leaning rice	AS53	Indica-leaning rice	AS72	Indica-leaning rice
AS16	Indica-leaning rice	AS35	Indica-leaning rice	AS54	Indica-leaning rice	AS73	Indica-leaning rice
AS17	Indica-leaning rice	AS36	Japonica-leaning rice	AS55	Indica-leaning rice	AS74	Indica-leaning rice
AS18	Indica-leaning rice	AS37	Indica-leaning rice	AS56	Indica-leaning rice	AS75	Japonica-leaning rice
AS19	Indica-leaning rice	AS38	Indica-leaning rice	AS57	Indica-leaning rice	AS76	Japonica-leaning rice
