# Supplementary material for: Phenotypic Variation Characterization and Multiple-Method Analysis of Drought Resistance in Rice at the Germination Stage
Source: Plants (Basel). 2026 Oct 20;15(20):3220. doi: 10.3390/plants15203220 (PMC13708856; doi:10.3390/plants15203220)
Supplement: Supplementary file 1 [file plants-15-03220-s001.zip › Supplementary Table S6.pdf]

Supplementary Table S6.The Spearman's rank correlation coefficient calculated from the AvgDGT and CDR

	CDR	AvgDGT
CDR	1	0.76
AvgDGT	0.76	1

P<0.01
