# Supplementary material for: Phenotypic Variation Characterization and Multiple-Method Analysis of Drought Resistance in Rice at the Germination Stage
Source: Plants (Basel). 2026 Oct 20;15(20):3220. doi: 10.3390/plants15203220 (PMC13708856; doi:10.3390/plants15203220)
Supplement: Supplementary file 1 [file plants-15-03220-s001.zip › Supplementary Table S5.pdf]

Summary Table S5. Drought resistance classification and reduction rate performance of rice.

No.	Reduction rate	Drought resistance level	No.	Reduction rate	Drought resistance level	No.	Reduction rate	Drought resistance level	No.	Reduction rate	Drought resistance level
AS1	0.26	2	AS20	0.20	1	AS39	0.46	3	AS58	0.46	3
AS2	0.36	2	AS21	0.45	3	AS40	0.58	3	AS59	0.28	2
AS3	0.25	1	AS22	0.46	3	AS41	0.77	4	AS60	0.24	1
AS4	0.28	2	AS23	0.49	3	AS42	0.45	3	AS61	0.24	1
AS5	0.46	3	AS24	0.55	3	AS43	0.83	4	AS62	0.25	2
AS6	0.29	2	AS25	0.26	2	AS44	0.73	4	AS63	0.23	1
AS7	0.26	2	AS26	0.42	3	AS45	0.55	3	AS64	0.23	1
AS8	0.28	2	AS27	0.48	3	AS46	0.84	4	AS65	0.22	1
AS9	0.23	1	AS28	0.44	3	AS47	0.29	2	AS66	0.46	3
AS10	0.25	1	AS29	0.36	2	AS48	0.67	4	AS67	0.56	3
AS11	0.33	2	AS30	0.39	2	AS49	0.32	2	AS68	0.47	3
AS12	0.28	2	AS31	0.73	4	AS50	0.67	4	AS69	0.52	3
AS13	0.27	2	AS32	0.49	3	AS51	0.33	2	AS70	0.47	3
AS14	0.30	2	AS33	0.51	3	AS52	0.35	2	AS71	0.59	3
AS15	0.28	2	AS34	0.53	3	AS53	0.28	2	AS72	0.48	3
AS16	0.36	2	AS35	0.47	3	AS54	0.27	2	AS73	0.64	3
AS17	0.35	2	AS36	0.67	4	AS55	0.30	2	AS74	0.68	4
AS18	0.21	1	AS37	0.38	2	AS56	0.36	2	AS75	0.80	4
AS19	0.23	1	AS38	0.45	3	AS57	0.39	2	AS76	0.74	4
