# Supplementary material for: Phenotypic Variation Characterization and Multiple-Method Analysis of Drought Resistance in Rice at the Germination Stage
Source: Plants (Basel). 2026 Oct 20;15(20):3220. doi: 10.3390/plants15203220 (PMC13708856; doi:10.3390/plants15203220)
Supplement: Supplementary file 1 [file plants-15-03220-s001.zip › Supplementary Table S4.pdf]

Supplementary Table S4. Statistical analysis of drought resistance during germination stage using GC

NO.	GC of each indicator									GC	Ranking
	GP	GR	BI	VI	GDRI	PH	RL	RSR	SRWC		
AS1	2.00	2.00	2.00	3.00	2.00	2.00	3.00	3.00	2.00	0.5	29
AS2	2.00	2.00	2.00	2.00	2.00	2.00	2.00	3.00	1.00	0.6	14
AS3	1.00	2.00	2.00	1.00	2.00	2.00	1.00	1.00	1.00	0.8	3
AS4	1.00	2.00	2.00	2.00	2.00	2.00	3.00	3.00	3.00	0.5	22
AS5	1.00	2.00	2.00	3.00	2.00	3.00	3.00	3.00	4.00	0.4	39
AS6	1.00	2.00	2.00	2.00	2.00	2.00	3.00	3.00	2.00	0.6	18
AS7	2.00	2.00	2.00	2.00	2.00	2.00	2.00	3.00	2.00	0.6	19
AS8	2.00	2.00	2.00	2.00	2.00	2.00	3.00	3.00	2.00	0.5	23
AS9	1.00	2.00	1.00	2.00	1.00	2.00	2.00	3.00	3.00	0.7	10
AS1	1.00	2.00	2.00	2.00	1.00	2.00	2.00	3.00	2.00	0.7	11
AS1	3.00	2.00	2.00	1.00	2.00	3.00	1.00	1.00	3.00	0.6	15
AS1	2.00	2.00	2.00	1.00	2.00	3.00	1.00	1.00	3.00	0.7	12
AS1	2.00	2.00	3.00	2.00	3.00	2.00	2.00	2.00	2.00	0.5	24
AS1	2.00	2.00	2.00	2.00	2.00	2.00	3.00	3.00	2.00	0.5	25
AS1	3.00	2.00	2.00	2.00	2.00	2.00	2.00	2.00	2.00	0.6	20
AS1	3.00	2.00	2.00	2.00	2.00	2.00	2.00	2.00	2.00	0.6	21
AS1	3.00	2.00	2.00	2.00	2.00	2.00	2.00	2.00	3.00	0.5	26
AS1	1.00	2.00	1.00	1.00	1.00	2.00	2.00	2.00	2.00	0.8	5
AS1	1.00	2.00	1.00	1.00	2.00	2.00	2.00	2.00	2.00	0.7	7
AS2	1.00	2.00	1.00	1.00	1.00	2.00	2.00	2.00	2.00	0.8	6
AS2	3.00	2.00	3.00	3.00	3.00	2.00	2.00	3.00	3.00	0.4	46
AS2	3.00	2.00	3.00	3.00	3.00	2.00	3.00	3.00	4.00	0.3	56
AS2	3.00	2.00	3.00	3.00	3.00	2.00	3.00	3.00	3.00	0.4	52
AS2	3.00	2.00	2.00	2.00	2.00	2.00	3.00	3.00	3.00	0.5	35
AS2	2.00	2.00	2.00	2.00	2.00	2.00	3.00	3.00	3.00	0.5	30
AS2	3.00	2.00	3.00	3.00	3.00	3.00	3.00	3.00	4.00	0.3	61
AS2	3.00	2.00	3.00	3.00	3.00	3.00	3.00	3.00	4.00	0.3	62
AS2	3.00	2.00	3.00	3.00	3.00	2.00	2.00	2.00	3.00	0.4	40
AS2	3.00	2.00	2.00	2.00	2.00	2.00	2.00	2.00	3.00	0.5	27
AS3	3.00	2.00	2.00	2.00	2.00	2.00	3.00	3.00	3.00	0.5	35
AS3	3.00	2.00	3.00	3.00	3.00	3.00	3.00	2.00	4.00	0.3	57
AS3	4.00	2.00	3.00	3.00	3.00	3.00	2.00	1.00	3.00	0.4	47

AS3	3.00	2.00	3.00	3.00	3.00	3.00	3.00	2.00	4.00	0.3	58
AS3	3.00	2.00	3.00	3.00	3.00	3.00	3.00	3.00	4.00	0.3	63
AS3	3.00	2.00	3.00	3.00	3.00	3.00	3.00	2.00	3.00	0.4	54
AS3	3.00	3.00	3.00	3.00	3.00	4.00	3.00	2.00	4.00	0.3	67
AS3	2.00	2.00	2.00	2.00	2.00	2.00	2.00	2.00	2.00	0.6	16
AS3	3.00	2.00	3.00	3.00	3.00	2.00	2.00	3.00	2.00	0.4	41
AS3	2.00	2.00	2.00	3.00	2.00	2.00	3.00	4.00	4.00	0.4	48
AS4	3.00	2.00	3.00	3.00	3.00	3.00	3.00	2.00	1.00	0.4	42
AS4	4.00	4.00	4.00	4.00	4.00	4.00	3.00	3.00	2.00	0.1	72
AS4	3.00	3.00	3.00	3.00	3.00	4.00	3.00	2.00	2.00	0.3	59
AS4	4.00	4.00	4.00	4.00	4.00	4.00	4.00	3.00	3.00	0.0	75
AS4	4.00	4.00	3.00	4.00	4.00	4.00	4.00	3.00	2.00	0.1	73
AS4	4.00	4.00	3.00	4.00	4.00	4.00	3.00	1.00	2.00	0.2	68
AS4	4.00	4.00	4.00	4.00	4.00	4.00	4.00	1.00	1.00	0.2	69
AS4	4.00	4.00	3.00	4.00	4.00	4.00	3.00	2.00	2.00	0.2	70
AS4	4.00	4.00	4.00	4.00	4.00	4.00	4.00	4.00	2.00	0.0	76
AS4	4.00	4.00	4.00	4.00	4.00	4.00	3.00	1.00	2.00	0.2	71
AS5	4.00	4.00	4.00	4.00	4.00	4.00	4.00	3.00	1.00	0.1	74
AS5	2.00	2.00	2.00	3.00	2.00	3.00	3.00	3.00	3.00	0.4	43
AS5	2.00	2.00	2.00	2.00	2.00	1.00	3.00	3.00	1.00	0.6	17
AS5	2.00	2.00	2.00	2.00	2.00	2.00	3.00	3.00	3.00	0.5	31
AS5	2.00	2.00	1.00	2.00	2.00	3.00	3.00	3.00	3.00	0.5	32
AS5	2.00	2.00	1.00	2.00	1.00	3.00	3.00	3.00	3.00	0.5	28
AS5	2.00	2.00	2.00	2.00	2.00	3.00	3.00	2.00	3.00	0.5	33
AS5	2.00	2.00	1.00	3.00	2.00	3.00	3.00	4.00	2.00	0.5	37
AS5	2.00	2.00	2.00	3.00	2.00	2.00	3.00	3.00	3.00	0.5	38
AS5	2.00	2.00	1.00	2.00	2.00	3.00	3.00	3.00	3.00	0.5	34
AS6	1.00	2.00	1.00	1.00	1.00	1.00	1.00	2.00	1.00	0.9	1
AS6	1.00	2.00	2.00	2.00	1.00	2.00	3.00	3.00	1.00	0.7	13
AS6	2.00	2.00	2.00	2.00	1.00	1.00	2.00	3.00	1.00	0.7	9
AS6	1.00	2.00	1.00	1.00	1.00	1.00	2.00	3.00	1.00	0.8	4
AS6	1.00	2.00	1.00	2.00	1.00	2.00	2.00	3.00	1.00	0.7	8
AS6	1.00	1.00	1.00	1.00	1.00	2.00	2.00	2.00	1.00	0.8	2
AS6	3.00	2.00	2.00	3.00	2.00	4.00	3.00	3.00	1.00	0.4	44
AS6	3.00	2.00	3.00	3.00	3.00	3.00	3.00	3.00	2.00	0.4	54
AS6	3.00	2.00	3.00	3.00	3.00	2.00	3.00	3.00	2.00	0.4	49
AS6	4.00	2.00	3.00	3.00	3.00	2.00	2.00	3.00	1.00	0.4	45
AS7	4.00	2.00	3.00	3.00	3.00	2.00	3.00	3.00	3.00	0.3	60
AS7	4.00	2.00	3.00	3.00	3.00	2.00	3.00	3.00	2.00	0.4	55
AS7	3.00	2.00	3.00	3.00	3.00	2.00	3.00	3.00	2.00	0.4	50

AS7	3.00	2.00	3.00	3.00	3.00	2.00	2.00	3.00	3.00	0.4	51
AS7	4.00	2.00	3.00	3.00	3.00	3.00	3.00	3.00	3.00	0.3	64
AS7	3.00	2.00	3.00	3.00	3.00	2.00	3.00	4.00	4.00	0.3	65
AS7	4.00	2.00	3.00	3.00	3.00	3.00	3.00	3.00	3.00	0.3	66

GP represents germination potential, GRrepresents germination rate, BI represents bud index, VI represents vitality index, SRWC represents seedling relative water content, GDRI represents germination drought resistance index, RL represents root length, PH represents plant height, RSR represents root-to-shoot ratio, GC represents grading coefficient.
